# Evidence That Calebin A, a Component of *Curcuma Longa* Suppresses NF-κB Mediated Proliferation, Invasion and Metastasis of Human Colorectal Cancer Induced by TNF-β (Lymphotoxin)

**DOI:** 10.3390/nu11122904

**Published:** 2019-12-01

**Authors:** Constanze Buhrmann, Bastian Popper, Ajaikumar B. Kunnumakkara, Bharat B. Aggarwal, Mehdi Shakibaei

**Affiliations:** 1 Musculoskeletal Research Group and Tumor Biology, Chair of Vegetative Anatomy, Institute of Anatomy, Faculty of Medicine, Ludwig-Maximilian-University Munich, Pettenkoferstrasse 11, D-80336 Munich, Germany; constanze.buhrmann@med.uni-muenchen.de; 2 Biomedical Center, Core facility animal models, Ludwig-Maximilian-University Munich, D-82152 Martinsried, Germany; Bastian.Popper@bmc.med.lmu.de; 3 Institute of Pathology, School of Medicine, Technical University of Munich, D-81675 Munich, Germany; 4 Cancer Biology Laboratory & DBT-AIST International Laboratory for Advanced Biomedicine (DAILAB), Department of Biosciences & Bioengineering, Indian Institute of Technology Guwahati, Assam 781039, India; ajai78@gmail.com; 5 Inflammation Research Center, San Diego, CA 92126, USA; bbaggarwal@gmail.com

**Keywords:** Calebin A, Curcumin, Colorectal Cancer, TNF-β (Lymphotoxin), NF-κB, Metastasis

## Abstract

Objective: Natural polyphenol Calebin A has been recently discovered as a novel derivate from turmeric with anti-cancer potential. Pro-inflammatory cytokine TNF-β (lymphotoxin α) is a stimulant for cancer cell malignity via activation of NF-κB pathway, also in colorectal cancer (CRC). Here, we investigated the potential of Calebin A to suppress TNF-β-induced NF-κB signalling in CRC. Materials and Methods: Three distinct CRC cell lines (HCT116, RKO, SW480) were treated in monolayer or 3-dimensional alginate culture with TNF-β, Calebin A, curcumin, BMS-345541, dithiothreitol (DTT) or antisense oligonucleotides-(ASO) against NF-κB. Results: Calebin A suppressed dose-dependent TNF-β-induced CRC cell vitality and proliferation in monolayer culture. Further, in alginate culture, Calebin A significantly suppressed TNF-β-enhanced colonosphere development, as well as invasion and colony formation of all three CRC cell lines investigated. Calebin A specifically blocked TNF-β-induced activation and nuclear translocation of p65-NF-κB, similar to curcumin (natural NF-κB inhibitor), BMS-345541 (specific IKK inhibitor) and ASO-NF-κB. Moreover, Immunofluorescence and Immunoblotting showed that Calebin A, similar to curcumin or BMS-345541 suppressed TNF-β-induced activation and nuclear translocation of p65-NF-κB and the transcription of NF-κB-promoted biomarkers associated with proliferation, migration and apoptosis, in a dose- and time-dependent manner. Those findings were potentiated by the specific treatment of extracted nuclei with DTT, which abrogated Calebin A-mediated nuclear p65-NF-κB-inhibition and restored p65-NF-κB-activity in the nucleus. Conclusion: Overall, these results demonstrate, for the first time, that multitargeted Calebin A has an anti-cancer capability on TNF-β-induced malignities through inhibitory targeting of NF-κB activation in the cytoplasm, as well as by suppressing the binding of p65-NF-κB to DNA.

## 1. Introduction

To date, a growing population worldwide faces an increased risk of developing colorectal cancer (CRC) [1]. Additionally, clinical difficulties arise with treatment of CRC, as conventional therapies are not only expensive, but patients frequently develop chemoresistance [2,3]. It is projected that, by the year 2035, the number of deaths directly related to CRC will rise to 60%−70% worldwide [1], demonstrating the urgent need for novel, prophylactic therapeutic strategies.

DNA damage, inflammation, and oxidative stress all stimulate, enhance and drive cancer development and progression [4]. It is well accepted now that low-grade chronic inflammation is a major cause for a number of diseases, including cancer [5,6]. The transcription factor nuclear factor-kappaB (NF-κB), as a common responder to varied stress stimuli, leading to chronic low grade inflammation, plays a key role here [7,8]. This master regulator of inflammation is located as an inactive form in the cytoplasm and, after activation, shuttles to the nucleus. Activation of NF-κB family members (p50/ NF-κB1, p52/ NF-κB2, c-Rel, p65/RelA and RelB) is tightly regulated by cytoplasmic inhibitory IκB family proteins [9,10]. The phosphorylation and activation of IκBs, in turn, is modulated by IκB kinase (IKK) complexes (IKKα, IKKβ, IKKγ) [11].

NF-κB is activated by various cytokines, pathogens or growth factors [8,11]. Previous studies have shown that TNF-β (lymphotoxin), a member of the tumor necrosis factor family, induces inflammatory effects in CRC cells with a similar potency to TNF-α [12,13]. TNF-β is the closest structural homologue to TNF-α and was already discovered in the mid-1980s [14,15]. In previous studies, our group could show that, in CRC cells, TNF-β activates the NF-κB signaling pathway, inducing cancer cell proliferation, invasion and up-regulating genes connected with metastasis, promotes epithelial-to-mesenchymal-transition, stimulates its own expression and further promotes expression of TNF-α [12,13].

As state of the art, standard protocol chemotherapies have severe side effects, are high-cost, and not affordable by all, the need arises for safe, efficacious and less expensive alternatives. Interestingly, to date, around 49% of actual cancer drugs are directly derived from natural substances [16]. Furthermore, as nutraceuticals such as derivatives from turmeric are already regularly consumed by the world population [17], and, for some, effectiveness in cancer treatment has been established, it is desirable to further investigate the enormous potential nature offers [18,19].

Turmeric (*Curcuma longa L*. Zingiberaceae) has several bioactive components, which have been shown to possess anti-cancer properties, the best known of which is curcumin (natural NF-κB inhibitor) [19,20,21]. Recently, Calebin A [(3E)-4-(4-hydroxy-3-methoxyphenyl)-2-oxobut-3-en-1-yl(2E)-3-(4-hydroxy-3-methoxyphenyl) -prop-2-enoate], a novel component derived from turmeric, first isolated at the beginning of the century, has come to attention [22,23]. Calebin A does not belong to the larger group of curcuminoids, which are considered the main active compounds found in turmeric [24], but to the small fraction of non-curcuminoides [25]. The content of Calebin A in turmeric extract ranges approximately around 0.001% [23,26]. Previously, Calebin A has been shown to possess anti-cancer properties [27], suppress adipogenesis [28], down-regulate osteoclastogenesis [29] and protect neuronal cells from beta-amyloid insult [22]. Further, Calebin A has been shown to exhibit its anti-cancer properties by inducing apoptosis, inhibiting cell growth, and to modulate mitogen-activated protein kinases (MAPK) c-Jun N-terminal kinase (JNK), extracellular signal-regulated kinase (ERK) and protein kinase of 38 kDa (p38) in gastric cancer and neurofibroma [30,31]. More interestingly, this effect was also observed in chemoresistant cells, underlining the immense potential of Calebin A as a chemopreventive/chemosensitivity agent to improve and enhance standard therapeutic approaches. Recently, it was shown that Calebin A exhibits anti-oxidant activity and inhibits histone acetyltransferase (HAT) activity and further mediates its anti-cancer effects by inhibiting P300/CBP-associated factor (PCAF) [32]. A plethora of nutraceuticals have also been shown to prevent NF-κB activation by the inhibition of IKK activity, phosphorylation and degradation of IκBα, nuclear translocation or binding of p65-NF-κB to the DNA [5]. Interestingly, a recent study on different cell lines has shown that Calebin A also targets the NF-κB pathway, thereby suppressing cell survival and expression of NF-κB-regulated gene end-products, leading to the inhibition of cell growth and chemosensitization [27].

The potential modulating role of Calebin A in TNF-β-induced NF-κB mediated inflammatory signaling in colon cancer cells is unknown. Therefore, in the present study we investigated, for the first time, the efficacy of Calebin A on suppressing TNF-β-induced NF-κB mediated malignity in CRC cells in vitro.

## 2. Materials and Methods

### 2.1. Cell Lines

The colon cancer cell lines HCT116 and SW480 were obtained from the European Collection of Cell Cultures (Salisbury, UK). The colon cancer cell line RKO was from the American Type Culture Collection. Cells were cultured in a humidified incubator (37 °C, 5% CO_2_) with normal cell culture growth medium containing 10% FCS, as described in detail before [33].

### 2.2. Reagents and Antibodies

Calebin A was given as a kind gift by Sabinsa Corporation, East Windsor, NJ, USA. Alginate, Curcumin, BMS-345541, dithiothreitol (DTT), anti-β1-Integrin and anti-β-actin were purchased from Sigma (Munich, Germany), Epon was from Plano (Marburg, Germany). Stock solutions of DTT and BMS-345541 were prepared in PBS and further diluted in normal cell culture growth medium to obtain final concentrations. Calebin A and Curcumin were prepared in dimethylsulfoxide (DMSO) as a 5 mM stock solution and stored in small aliquots at −20 °C. During treatment, concentrations of DMSO did not exceed 0.1%. TNF-β was purchased from eBiosciences (Frankfurt, Germany) and additionally TNF-β (specific activity of 50 million U/mg) was given as a kind gift by Genetech (South San Francisco, CA, USA). Antibodies to p65-NF-κB, CXCR4, Caspase-3 and MMP-9 were from R&D Systems (Heidelberg, Germany). Ki-67 and secondary antibodies for fluorescence labelling were from Dianova (Hamburg, Germany).

### 2.3. Experimental Setup

CRC cell lines (HCT116, SW480, RKO) in monolayer and/or 3-dimensional alginate culture were investigated to elucidate the mechanism of Calebin A on suppressing TNF-β-induced inflammation and malignity in CRC. Prior to the experiments, cells were washed with serum-starved cell culture medium (only 3% FCS) and incubated for 1 h in a serum-starved medium. All experiments were performed in serum-starved medium.

### 2.4. Three-Dimensional Cell Culture

3D alginate culture presents a similar to vivo microenvironment superior to monolayer conditions and is very suitable for studying early events in tumor progression. CRC cells were re-suspended in alginate (2.5% in PBS) and this solution was added dropwise into a CaCl_2_-solution for the polymerization of alginate into stable beads, as previously described in detail [33,34,35].

### 2.5. Transfection with Antisense Oligonucleotides

For transient transfection with antisense/sense oligonucleotides (ASO/SO) targeting NF-κB, phosphorothioate-specific oligonucleotides (21-mer) were synthesized by Eurofins MWG Operon (Ebersberg, Germany). ASO sequence was 5′-gGAGATGCGCACTGTCCCTGGtC-3′ (corresponding to NF-κB/p65 subunit mRNA) and control SO sequence was 5′-gACCAGGGACAGTGCGCATCtC-3′. To protect oligonucleotides from cell nucleases, they were phosphorothioate-modified. Transfection was performed by using 10 µl/mL Lipofectin (Invitrogen) and 0.5 µM ASO/SO [36].

### 2.6. Cell Vitality Assay

CRC cell vitality and proliferation in monolayer culture were investigated by an MTT (3-(4,5-dimethylthiazol-2-yl)-2,5-diphenyltetrazolium bromide) assay and measured (Optical Density at 550 nm) using a revelation 96-well multiscanner plate ELISA reader (Biorad, Munich, Germany), as described in detail [34]. Each experiment was repeated at least three times and the obtained values were compared to the control, and statistically significant values with *p* < 0.05 were designated by an asterisk (*); *p* < 0.01 by two asterisks (**). For monolayer investigation, CRC cells were left untreated, treated with TNF-β (10 ng/mL), treated dose-dependently with Calebin A (0.01, 0.1, 1, 2, 5, 10 µM) alone and/or in combination with TNF-β for 48 h.

### 2.7. Proliferation and Migration Assay

The influence of Calebin A and TNF-β on the migration capacity of CRC cells was investigated, as previously described in detail [33,35]. CRC cells in alginate beads were left untreated, treated with Calebin A (0.1, 1, 2, 5 µM), TNF-β (10 ng/mL) alone, or Calebin A (2 µM) in combination with TNF-β, for 14 days. In the alginate beads, the CRC cells proliferated and formed spheroids. Around day 10, CRC cells in alginate bead culture migrated from the beads and formed new adhered colonies at the bottom of the petri dishes. On day 14, colonies were fixed in methanol, stained with toluidine blue and manually quantified under a light microscope (Zeiss, Germany). Each experiment was repeated at least three times and the obtained values were compared to the control, and statistically-significant values with *p* < 0.05 were designated by an asterisk (*); *p* < 0.01 by two asterisks (**).

### 2.8. Transmission Electron-Microscopy

CRC cells in alginate beads were left untreated, treated with Calebin A (0.1, 1, 2, 5 µM), or TNF-β (10 ng/mL) alone, or the indicated concentration of Calebin A (2 µM) in combination with TNF-β. Ultrastructural investigations were performed with a transmission electron-microscope (Jeol 1200 EXII, Akishima Tokyo, Japan (Institute of Pathology, Technical University of Munich, Germany), as described before [35]. In short, the alginate beads were fixed in Karnowsky fixative, post-fixated in 1% O_s_O_4_ solution, and finally embedded in Epon, and ultrathin sections prepared on a Reichert–Jung Ultracut E (Darmstadt, Germany).

### 2.9. Immunofluorescence Imaging

Monolayer CRC cells were left untreated, treated with Calebin A (5 µM), curcumin (5 µM), BMS-345541 (5 µM), TNF-β (10 ng/mL) or pre-treated for 1 h with the indicated concentration of Calebin A, curcumin or BMS-345541 by itself, followed by combination treatment with TNF-β for 4 h. CRC cells were fixed in methanol and stored at -20 °C. Immunofluorescence labelling was performed as previously described [33]. In short, primary antibodies to p65-NF-κB were diluted 1:100 and incubated overnight at 4 °C, rhodamine-coupled secondary antibodies (1:100) were incubated for 2 h at ambient temperature (AT). Nuclear staining was performed with DAPI (Sigma, Germany) and images captured with a Leica DM2000 microscope (Wetzlar, Germany).

### 2.10. Western Blot Analysis

Immunoblotting was performed as described in detail by Shakibaei et al. [35]. In short, CRC cells were lysed and total protein content was measured with serum bovine albumin as standard (bicinchinonic acid system, Uptima, France). Further, samples were reduced with 2-mercaptoethanol, protein content adjusted, and proteins separated with SDS-PAGE electrophoresis and transferred to a nitrocellulose (Mini-PROTEAN Tetra Cell System, Biorad, Munich, Germany). After blocking for 2 h, primary antibodies were incubated overnight and secondary alkaline phosphatase linked antibodies incubated for 2 h at AT. Finally, antibody-antigen complexes were visualized using nitroblue tetrazolium and 5-bromo-4-chloro-3-indoylphosphate (p-toluidine salt; Pierce, Rockford, Illinois USA).

Monolayer cultures of CRC were investigated for time and dose-dependent effects of Calebin A on TNF-β signaling pathway activation. For the time-dependent investigation, cells were left untreated, or treated with Calebin A (5 µM) alone or TNF-β (10 ng/mL) alone, and samples taken at 5, 10, 20, 40 and 60 min. In a subsequent approach, CRC cells were pre-treated for 1 h with Calebin A (5 µM) alone, followed by combination treatment with TNF-β (10 ng/mL) and samples taken at 5, 10, 20, 40 and 60 min. For dose-dependent investigation, CRC cells were left untreated, treated with TNF-β (10 ng/mL), pre-treated for 1 h with Calebin A (0.1, 1, 2, 5 µM) alone and/or followed by combination treatment with TNF-β (10 ng/mL) for 4 h. Additionally, to compare the effect on the activation of NF-κB, CRC cells were treated with Calebin A (5 µM), TNF-β (10 ng/mL), curcumin (5 µM), ASO/SO-NF-κB (0.5 µM/Lipofectin) and BMS-345541 (5 µM) for 4 h. In a subsequent approach, nuclear extracts were prepared to specifically investigate the effect of Calebin A on p65-NF-κB binding to the DNA in the nucleus. CRC cells in monolayer were left untreated and/or pre-treated for 4 h with TNF-β (10 ng/mL) and cytoplasm was extracted with a cytoplasmic extraction buffer and discarded. The remaining nuclei were left untreated and/or incubated for 1 h with Calebin A (5 µM), curcumin (5 µM) or DTT (5 mM) alone, or co-treated with Calebin A or curcumin and DTT. Finally, nuclear extracts were prepared for Western blotting to investigate the effects on nuclear NF-κB.

### 2.11. Statistical Evaluation

The results were evaluated by Wilcoxon–Mann–Whitney test. The data are demonstrated as mean + standard deviation or SEM, and were compared by one-way or a two-way ANOVA using SPSS Statistics, if the normality test passed (Kolmogorov–Smirnov test). Each experiment was performed at least three times. P-value of *p* < 0.05 was considered statistically significant.

## 3. Results

This study was performed to examine the anticancer potential of Calebin A and whether Calebin A suppresses the TNF-β-promoted p65-NF-κB activation and NF-κB-mediated gene products in colorectal cancer cells.

### 3.1. Calebin A Suppressed TNF-β-Promoted Proliferation in Different CRC Cells

The effect of Calebin A on the proliferation and vitality of the CRC cell lines HCT116, RKO and SW480 was investigated by the MTT method in monolayer culture, as described in Materials and Methods.

**Figure 1:**
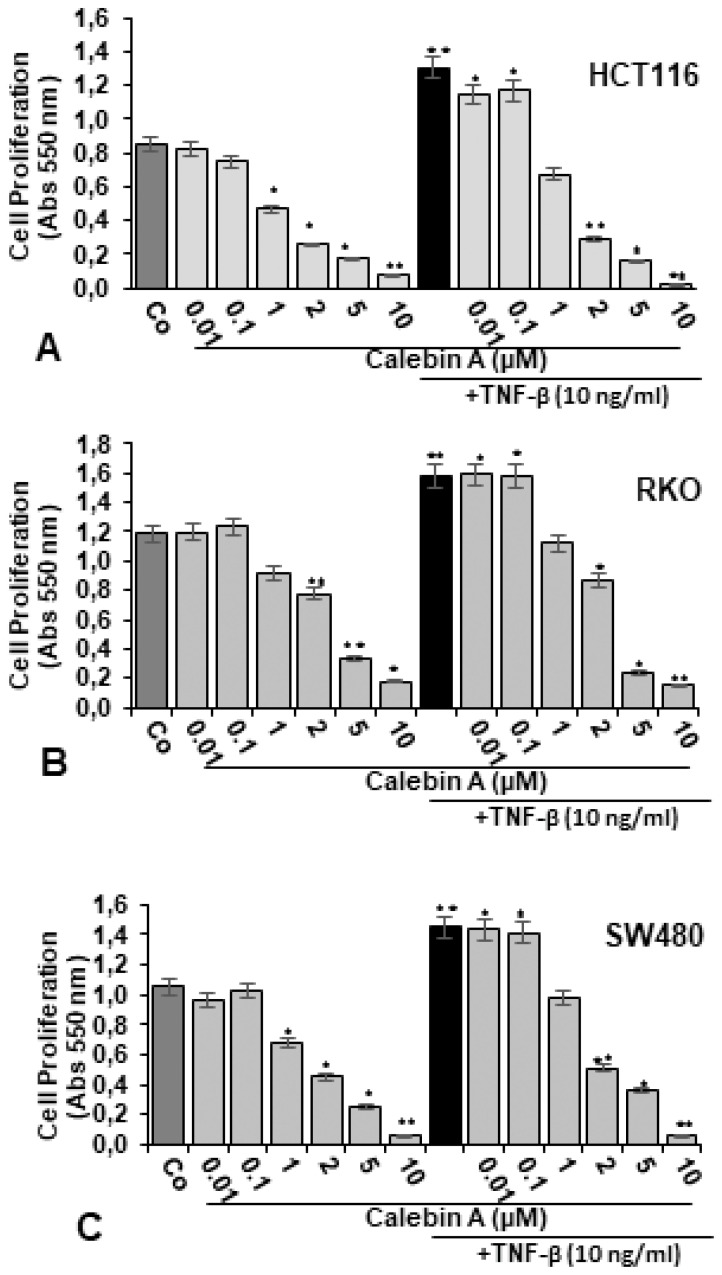
Effect of Calebin A alone and/or TNF-β on proliferation of different CRC cell lines in monolayer cultures. Serum-starved CRC cell lines, HCT116 (**A**), RKO (**B**), SW480 (**C**) in monolayer cultures were treated as described in Materials and Methods. The MTT assay was performed to evaluate the proliferation of CRC cells, as described in detail in Materials and Methods. All assays were performed at least three times. *p* < 0.05 (*) and *p* < 0.01 (**) indicate a significant difference compared to the control group.

In Monolayer, treatment with Calebin A alone did not significantly suppress cell viability and proliferation in all three CRC cell lines at low concentrations of 0.01 and 0.1 µM, compared to untreated control cultures (Figure 1A−C). However, higher concentrations of Calebin A dose-dependently significantly reduced cell proliferation in all three cell lines. Cell viability was suppressed in HCT116 by 43%, 66%, 78% and 88%, in RKO by 25%, 33%, 64% and 84%, in SW480 by 32%, 51%, 76% and 91% treated with Calebin A 1, 2, 5 and 10 µM, respectively (Figure 1A−C). This highlights clearly that the CRC cells exhibit different sensitivities to Calebin A treatment in monolayer: with IC50 for the HCT116 range at around 2 µM, whereas RKO and SW480 are less sensitive to Calebin A, with IC50 ranges of around 5 µM.

Treatment with TNF-β (10 ng/mL) alone significantly stimulated cell proliferation in all three CRC cells lines compared to untreated controls by 51% in HCT116, by 33% in RKO and by 41% in SW480 (Figure 1A-C). Co-treatment of TNF-β and Calebin A at low concentrations of 0.01 µM and 0.1 µM did not significantly block cell proliferation compared to TNF-β mono-treatment. However, interestingly, compared to TNF-β mono-treatment, co-treatment with TNF-β and increasing concentrations of Calebin A markedly blocked the TNF-β-induced proliferation effect on CRC cells (Figure 1A−C). A significant dose-dependent effect was observed in all three cell lines with a decrease in cell proliferation by 46%, 73%, 86% and 96% in HCT116, by 26%, 44%, 84% and 88% in RKO, and by 32%, 65%, 69% and 94% in SW480, treated with TNF-β (10 ng/mL) and Calebin A 1, 2, 5 and 10 µM, respectively, compared to TNF-β mono-treatment.

### 3.2. Calebin A Significantly Supresses TNF-β-Promoted Colonosphere Formation, Invasion and Stimulates Apoptosis of CRC Cells

**Figure 2:**
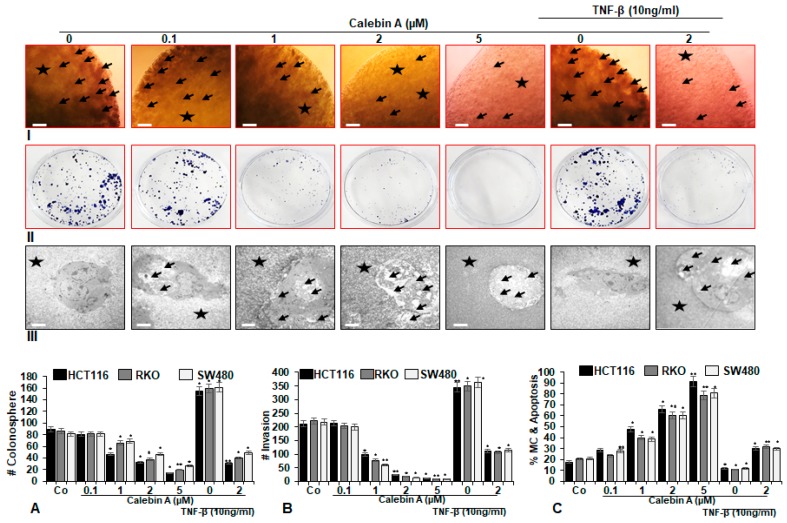
Effect of Calebin A alone and/or TNF-β on colonosphere, invasion/metastasis and apoptosis in CRC cells in 3D-alginate tumor cultures. Serum-starved CRC cell lines (HCT116, RKO, SW480) in alginate tumor cultures were treated as described in Materials and Methods. I: Light microscopic presentation of HCT116 (**A, I**), RKO (**A**), SW480 (**A**) cell lines grown in alginate beads culture (*), treated with Calebin A and/or TNF-β after 14 days. The amount of colonospheres (arrows) was quantified by counting 20 different microscopic fields. Magnification x24, scale bar = 0.2 mm in all cases. II: Invasive colonies of HCT116 (**B, II**), RKO (**B**), SW480 (**B**) cells, treated with Calebin A, were labelled with toluidine blue after 14 days of culture. The amount of invasive and adhered colonies to petri dishes was demonstrated and quantified by calculating all colonies under a light microscope (Zeiss, Germany). * *p* < 0.05, ** *p* < 0.01. III: Electron microscopic illustration of cell survival and apoptosis of HCT116 (**C, III**), RKO (**C**), SW480 (**C**) cells after treatment with Calebin A and/or in TNF-β-induced inflammatory microenvironment, as described in detail in Materials and Methods, for 14 days. Apoptotic bodies and mitochondrial changes (MC) (arrows) in alginate (*) were visible. Magnification, 5000x. Scale bar = 1 µm. To quantify MC and apoptosis in tumor cells, 100 cells from 20 microscopic fields were counted. Values were compared with the control and statistically significant values with *p* < 0.05 were designated by (*) and *p* < 0.01 were designated by (**).

We investigated the anti-tumorigenic effect of Calebin A in TNF-β-promoted inflammatory tumor microenvironment in 3D-alginate cultures for 14 days, as described in Materials and Methods. In control cultures of all CRC cells, HCT116 (Figure 2A,B, I–II), RKO, SW480 (Figure 2A,B), marked CRC cell proliferation, colonosphere development, invasion and colony formation was observed. In contrast to this, treatment with Calebin A markedly suppressed CRC cells, HCT116 (Figure 2A,B, I–II), RKO, SW480 (Figure 2A,B) proliferation, colonosphere formation, invasion and colony formation in a dose-dependent manner (Figure 2A,B, I–II). TNF-β alone markedly stimulated proliferation, colonosphere formation, substantially enhanced invasion and significantly increased new colony formation in all three cell lines (Figure 2A,B, I–II). Contrary to this, co-treatment with Calebin A suppressed these TNF-β-mediated effects, substantially blocking cell proliferation, colonosphere development, invasion and colony formation in all three CRC cell lines, HCT116 (Figure 2A,B, I–II), RKO, SW480 (Figure 2A,B). Statistical evaluation of Calebin A alone, or combinational treatment with TNF-β from all three CRC cell lines, further underlines these results (Figure 2A,B, I–II).

To further elucidate the impact of Calebin A on an ultrastructural level on the metastatic behaviour of CRC cells, we performed Transmission-Electron-Microscopy (TEM), as described in Materials and Methods. In control alginate bead cultures, cells proliferated and formed colonosphere aggregates, containing rounded cells with abundant mitochondria, multiple cell organells and intact nuclei in CRC cells, HCT116 (Figure 2C, III), RKO, SW480 (Figure 2C). However, as shown in Figure 2C, III, Calebin A treatment dose-dependently clearly stimulated apoptosis in CRC cells with significant increase in cellular signs of apoptosis (increase in apoptotic bodies, nuclear fragmentation, multiple vacuole development). Similar to control cultures, TNF-β-treated cells were vital, exhibited a rounded morphology with multiple cell organells and intact nuclei. Interestingly, the co-treatment of Calebin A and TNF-β, suppressed positive effects of TNF-β on CRC cell vitality and markedly stimulated apoptosis in CRC cells, HCT116 (Figure 2C, III), RKO, SW480 (Figure 2C). Statistical quantification of apoptotic cells in each sample further underlines the anti-apoptotic effect of Calebin A alone, or in combinational treatment with TNF-β, in all three CRC cell lines compared to control cultures (Figure 2C).

### 3.3. Calebin A, Similar to Curcumin and Specific IKK Inhibitor BMS-345541, Blocks TNF-β-Promoted Nuclear Translocation of p65-NF-κB to the Cell Nucleus in Different CRC Cell Lines

**Figure 3:**
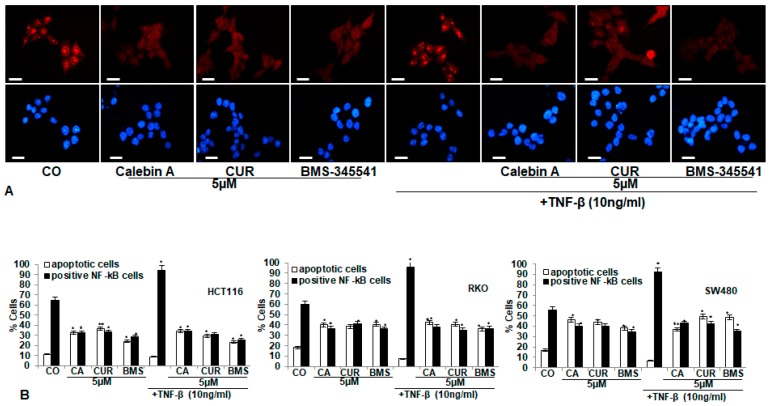
Effect of Calebin A or curcumin or specific IKK inhibitor BMS-345541 on TNF-β-promoted nuclear translocation of phospho-p65 to the cell nucleus in different CRC cell lines. A–B: Serum-starved CRC cell lines (HCT116, RKO, SW480) in monolayer cultures were treated as described in Materials and Methods. (**A**) HCT116 cells were labeled for p65-NF-κB by immunofluorescence and counterstained with DAPI. Magnification 600x; scale bar = 30 mm. (**B**) All experiments were performed at least in triplicate and evaluation of positively labelled nuclei and apoptotic cells was performed by counting 500−800 cells from 10 different microscopic fields. * *p* < 0.05, ** *p* < 0.01.

As TNF-induces stimulation and phosphorylation of NF-κB, a major transcription factor for enhancing colonosphere development, invasion and colony formation of CRC cells, we next investigated whether the suppressive effects of Calebin A in CRC cells were mediated by the inhibition of activation and nuclear translocation of NF-κB. CRC cell cultures (HCT116, RKO, SW480) were treated as described in Materials and Methods, and immunofluorescence labelling performed for p65-NF-κB. Control cultures revealed intense nuclear staining and weak cytoplasmatic staining in HCT116 (65%), RKO (60%) and SW480 (65%) cells (Figure 3). Treatment with TNF-β stimulated activation of NF-κB and nuclear staining markedly increased to 95% in HCT116, to 95.9% in RKO and to 93% in SW480. One hour’s pre-treatment with Calebin A, curcumin or BMS-345541 resulted in considerably lower amounts of nuclear staining in HCT116 to 34%, 35.7% and 30%, in RKO to 36%, 42% and 36%, in SW480 to 40.1%, 39% and 35%, respectively. Additionally, 1 h pre-incubation with Calebin A, curcumin or BMS-345541, followed by TNF-β co-treatment, markedly reduced nuclear staining in all CRC cell lines: in HCT116 to 36%, 33% and 30%, in RKO to 42%, 41% and 36% and in SW480 to 43%, 42% and 35%. These results indicate that, indeed, suppressive effects on proliferation and invasion by Calebin A, similar to curcumin, are mediated, at least in part, in CRC cells through the inhibition of the NF-κB signaling pathway. Further, Calebin A has the potential to significantly block tumorigenic stimulating effects induced by TNF-β.

### 3.4. Calebin A Supresses TNF-β-Promoted p65-NF-κB Activation Time-Dependently in All Three CRC Cell Lines

**Figure 4:**
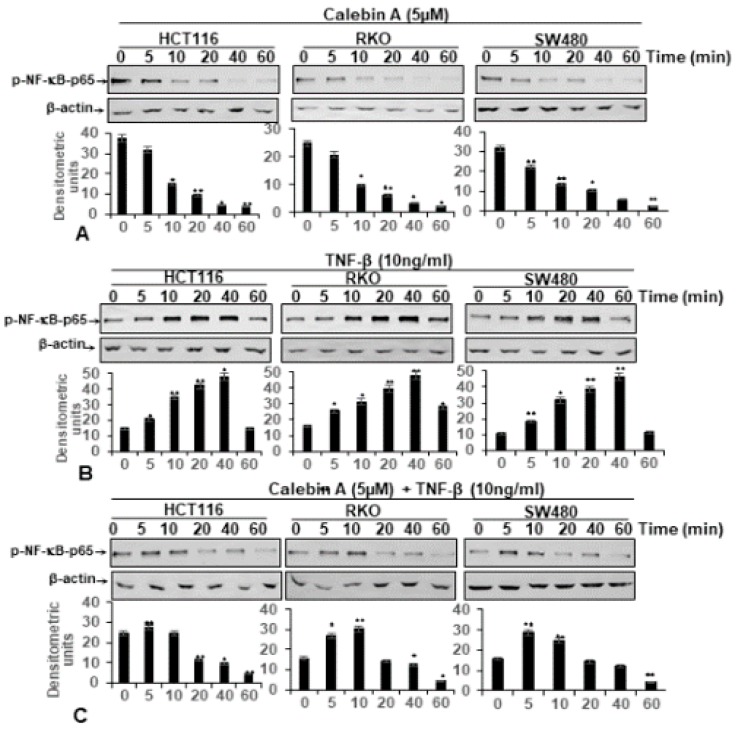
Calebin A suppressed TNF-β-induced p65-NF-κB phosphorylation, independent of CRC cell type. Time-dependent experiments of CRC cell lines (HCT116, RKO, SW480) in monolayer cultures treated with Calebin A alone (**A**), with TNF-β (**B**) or with Calebin A and TNF-β (**C**) were performed as described in Materials and Methods. Immunoblotting of whole cell lysates was performed for anti-phospho-p65. The results are shown from at least three independent experiments and the housekeeping protein β-actin served as an internal loading control. Densitometric evaluation was performed for phospho-p65 for all CRC cell lines. * *p* < 0.05, ** *p* < 0.01.

To further elucidate whether suppression of cell proliferation by Calebin A in CRC cells is mediated by targeting the NF-κB pathway, we performed western blot analysis of monolayer cultures, treated time-dependently with Calebin A, TNF-β or the combination as described in Materials and Methods. As shown in Figure 4B, compared to untreated control cultures, TNF-β markedly stimulates p65-NF-κB expression in HCT116, RKO and SW480 CRC cells in a time-dependent fashion. Interestingly, Calebin A alone or in combinational treatment with TNF-β is capable of significantly suppressing p65-NF-κB expression time-dependently in all three CRC cell lines (Figure 4A,C).

### 3.5. Calebin A Specifically Suppresses TNF-β-Induced p65-NF-κB Activation, Similar to Curcumin, ASO-NF-κB or BMS-345541 in CRC Cells

**Figure 5:**
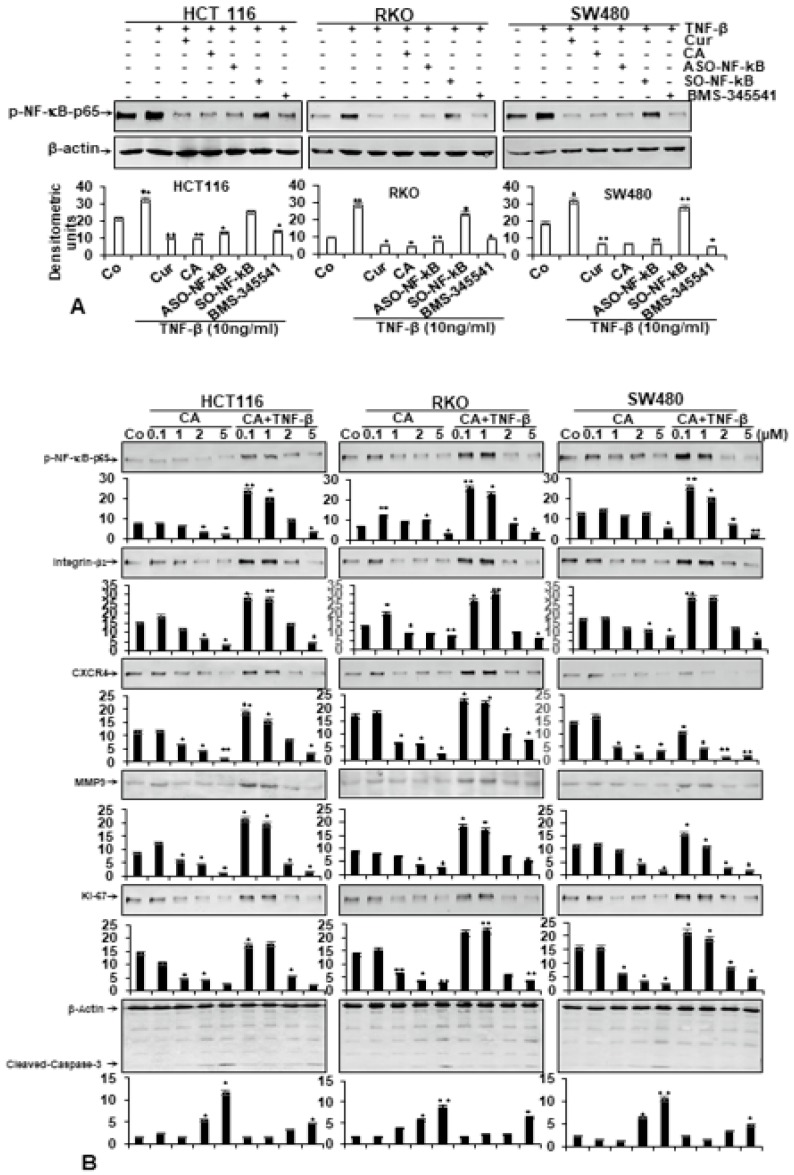
(**A**) Effect of Calebin A, curcumin, BMS-345541 and specific ASO against NF-κB on TNF-β-induced p65-NF-κB activation in different CRC cell lines. Serum-starved CRC cell lines (HCT116, RKO, SW480) in monolayer cultures were treated as described in Materials and Methods. Immunoblotting of whole cell lysates was performed for anti-phospho-p65. The results are shown from at least three independent experiments and the housekeeping protein β-actin served as an internal loading control. Densitometric evaluation was performed for phospho-p65 for all CRC cell lines. * *p* < 0.05, ** *p* < 0.01. (**B**) Effect of Calebin A on TNF-β-promoted p65-NF-κB activation and NF-κB-regulated gene products involved in proliferation and metastasis in a dose-dependent fashion in different CRC cell lines. Serum-starved CRC cell lines (HCT116, RKO, SW480) in monolayer cultures were treated as described in Materials and Methods. Immunoblotting of whole cell lysates was performed for anti-phospho-p65, anti-integrin-β1, anti-MMP-9, anti-Ki-67, anti-CXCR4 and anti-cleaved-caspase-3. The results are shown from at least three independent experiments and the housekeeping protein β-actin served as an internal loading control. Densitometric evaluation was performed for phospho-p65, β-Integrin, MMP-9, Ki-67, CXCR4 and cleaved-caspase-3 for all CRC cell lines. * *p* < 0.05, ** *p* < 0.01.

As NF-κB phosphorylation is the main first move in the signaling pathway of inflammation [37], we evaluated whether Calebin A coordinates TNF-β-promoted NF-κB activation. Further, we investigated whether Calebin A had a similar specific potential in suppressing TNF-β-induced NF-κB activation compared to curcumin (natural NF-κB inhibitor), ASO-NF-κB or BMS-345541 in CRC cells (HCT116, RKO, SW480). To elucidate the suppressive effects of Calebin A in TNF-β-promoted NF-κB signaling pathway, we investigated up-stream in the NF-κB signaling to show the effect of TNF-β-induced IκBα activation as a prior condition for p65-NF-κB phosphorylation. Furthermore, as IκBα phosphorylation and degradation require activation of IκB kinase (IKK), we evaluated the role of BMS-345541, a directed IKK suppressor, on TNF-β-induced IKK activity. Serum-starved CRC tumor cells were investigated for phosphorylated p65-NF-κB after treatment, as indicated in Materials and Methods (Figure 5A). Western blotting findings showed that TNF-β promoted phospho-p65 phosphorylation in all three CRC cell lines. Immunoblotting results in Figure 5A, highlighting that Calebin A has the potential to suppress TNF-β-induced p65-NF-κB-activation with the same intensity as curcumin, ASO-NF-κB or BMS-345541 in CRC cells. The quantification of blots highlights the immense potential of Calebin A as an anti-tumorigenic agent in CRC cells by specifically targeting NF-κB. All together, these findings were in accordance with the suppression of p65-NF-κB observed by immunofluorescence results. These results further indicate that Calebin A may play an essential role in the p65-NF-κB activation signaling pathway. Furthermore, the suppression effect of Calebin A on p65-NF-κB was not cell type-specific.

### 3.6. Calebin A Suppresses TNF-β-Promoted p65-NF-κB Activation and NF-κB-Dependent Gene Products Involved in Metastasis, Migration and Apoptosis of CRC Cells

To examine the basic mechanism of how Calebin A blocks TNF-β-promoted malignancy of CRC cells, we evaluated whether the effects of Calebin A on CRC cells in TNF-β-induced pro-inflammatory tumor microenvironments was associated with the suppression of proinflammatory transcription factor NF-κB phosphorylation and NF-κB-regulated gene proteins linked with tumor metastasis. Moreover, it has been shown that proinflammatory cytokines basically mediate their effects by activation of NF-κB, modulating the expression of inflammatory proteins associated in migration and metastasis [38,39]. Serum-starved CRC cells (HCT116, RKO, SW480) were either left untreated or treated as described in Materials and Methods. Next, we examined, as illustrated in Figure 5B, the expression of the p65-NF-κB and NF-κB-dependent gene proteins that are linked to invasion (MMP-9), metastasis (CXCR4, β1-integrin) and proliferation (Ki-67). The results of western blotting demonstrated a basal expression of the above-mentioned proteins expression in untreated cultures of CRC cells and this expression was extensively raised in the presence of TNF-β. In opposite to this, immunoblotting results demonstrated that Calebin A, alone or in combination with TNF-β, substantially suppressed the mentioned proteins’ expression in a dose-dependent fashion in all CRC cell lines. Together, these results indicate the important role of Calebin A in modulating TNF-β-induced, NF-κB-regulated tumor metastasis-promoting gene products in CRC cells.

Next, CRC cell lines were treated as described above, and subjected to immunoblotting to detect cleaved-caspase-3. As shown in Figure 5B, TNF-β had no effect on caspase-3 cleavage. In contrast, Calebin A, alone or in combination with TNF-β, up-regulated the cleavage of caspase-3 in a dose-dependent fashion (Figure 5B).

These findings support the MTT and electron microscopic results and indicate that Calebin A inhibits TNF-β-induced p65-NF-κB phosphorylation and NF-κB-activated pro-inflammatory, proliferative and migration gene expression in CRC cells. Furthermore, these data suggest further that the anti-carcinogenic effects of Calebin A are, in part, connected through the up-stream inhibition of activation of the p65-NF-κB pathway.

### 3.7. Calebin A Disrupts the Interaction of p65-NF-κB to DNA and DTT Suppresses this Binding

**Figure 6:**
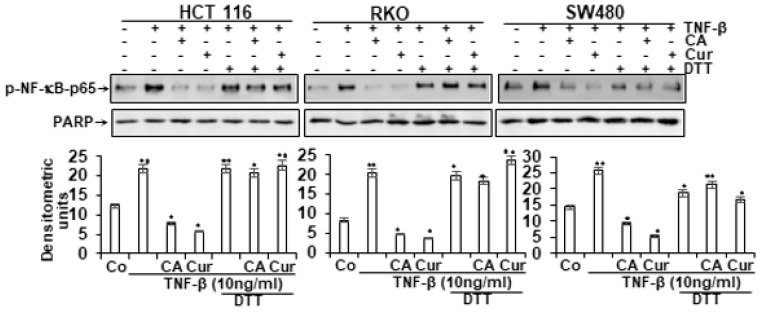
Effect of DTT on the inhibitory effect of Calebin A, and/or curcumin in TNF-β-induced p65-NF-κB binding to DNA. Extracted nuclei of TNF-β-treated CRC cell lines were co-treated with Calebin A (CA), and/or curcumin in the absence or presence of DTT and nuclear extracts then immunoblotted for p65-NF-κB activation. The results are demonstrated from at least three independent assays and the housekeeping protein β-actin served as an internal loading control. Densitometric evaluation was performed for phospho-p65 for all CRC cell lines. * *p* < 0.05, ** *p* < 0.01.

A large body of evidence has previously reported that several inhibitors of the p65-NF-κB transcription factor compete for the binding of p65 to DNA and the cysteine residues in the p65 subunit are responsible for its interaction with DNA [40,41,42,43,44,45,46]. Thus, we examined if a reduction in cysteine residues by DTT (dithiothreitol) in p65-NF-κB might influence the interaction of p65-NF-κB to the DNA binding in the presence or absence of Calebin A. Interestingly, we showed that the inhibition of Calebin A on p65-NF-κB interaction to DNA was prevented by DTT in all three distinct CRC cell lines (HCT116, RKO, SW480) (Figure 6). This finding indicates that Calebin A suppresses the binding of p65-NF-κB to DNA, suggesting it may be one of the primarily (essential) molecular mechanisms of Calebin A, as it blocks p65-NF-κB activation.

## 4. Discussion

The main object of this investigation was to evaluate the potency of Calebin A, an ingredient of turmeric, which is a pharmacologically safe natural agent and used comprehensively for the treatment of disease. We investigated the tumor suppressive effect of Calebin A on the malignity potential of three different colorectal cancer (CRC) cell lines (HCT116, SW480 and RKO), as all three cell lines are derived from primary tumors of three distinct patients (HCT116 and RKO from colorectal carcinomas and SW480 from colorectal adenocarcinoma) and exhibit different genetic features and mutations in cancer critical genes (microsatillite stability/instability, KRAS, RAF, PIk3CA and TP53) [47]. In this study, we examined whether Calebin A has the necessary properties to modulate TNF-β-promoted malignancy of CRC cells through the modulation of proinflammatory transcription factor NF-κB signaling pathway and to determine, at least in part, the mechanisms of the Calebin A molecular signaling during carcinogenesis, because they are still poorly understood.

Turmeric extract (*curcuma*) has been used for centuries in traditional Asian and Indian medicine and is, to date, an important ingredient in Asian and Indian cuisine [39,48]. The most intensively studied polyphenolic component of turmeric, curcumin, makes up around 2%−5% of total turmeric and has been shown in numerous studies to exhibit anti-inflammatory and anti-tumorigenic effects [24]. Recent studies have indicated numerous other chemical ingredients in turmeric next to curcumin with pharmacological potential, of which Calebin A seams very promising, with significant anti-tumor properties [22,24,25].

In this report, we showed that Calebin A blocked the TNF-β-promoted proliferation, colonosphere formation, invasion/metastasis of different CRC cell lines (HCT116, RKO and SW480) in monolayer or 3D-alginate cultures. Furthermore, Calebin A down-modulated the TNF-β-inhibited apoptosis in all CRC cell lines. Recently, similar to our findings, Calebin A was shown to effectively suppress dose-dependent proliferation of a different range of cancer cells, including myeloid leukemia (KBM-5), squamous cell carcinoma (SCC4), myeloma (U266 and MM.1S) and CRC cells (HCT116) [27]. Calebin A has been described by Kim and Kim [23] as a novel non-curcuminoid found in *curcuma species* such as turmeric (*curcuma longa*), where it makes up of around 0.001% of the total extract. Recent studies on Calebin A have further shown its vast pharmacological potential, and it exhibits anti-tumorigenic activity, suppresses adipogenesis and osteoclastogenesis and protects neurons from β-Amyloid insults [22,27,28,29,31]. Interestingly, it has been previously shown that TNF-β-induced CRC proliferation can be suppressed by other natural substances such as resveratrol and curcumin [13]. Indeed, curcumin has been shown to inhibit tumor cell proliferation, migration and metastasis in multiple tumors [49,50]. Thereby, it modulates a large range of intracellular signalling pathways, including PI3K/Akt, Wnt/β-catenin, JAK/STAT, p53 and NF-κB pathway [19,49]. Additionally, it has been previously shown that curcumin enhances the chemosensitivity of CRC to chemotherapeutics through regulation of MiR-34a and MiR-27a, activation of the MMR-repair system, influencing tumor–microenvironment cross-talk and suppression of NF-κB pathway activation [35,51,52,53,54]. This underlines that specific suppression of tumor-enhancing TNF-β stimulation not only bears the potential of Calebin A for suppressing CRC proliferation alone, but in combination with other natural substances as well, for even better results.

Additionally, we show for the first time that, in three-dimensional alginate culture in vitro, Calebin A suppressed TNF-β-stimulated proliferation, colonosphere formation, invasion, and enhanced apoptosis. The three-dimensional alginate culture provides an excellent microenvironment to study the early stages of tumor development, such as colonosphere formation, invasion, migration activity and colony formation of tumor cells [35]. Here, we demonstrate that Calebin A treatment dose-dependently markedly suppresses early development stages of tumor malignity. More importantly, this effect of Calebin A could also be observed in a pro-inflammatory, stimulated TNF-β-environment.

We have shown that Calebin A clearly blocked several stages to suppress TNF-β-promoted NF-κB activation in different CRC cells. Indeed, it has been previously indicated that Calebin A has anti-proinflammatory and anti-carcinogenic qualities, and these properties are linked to the proinflammatory transcription factor NF-κB signaling pathway [27,29,30]. Moreover, it has been shown that the proinflammatory transcription factor NF-κB pathway is required for, and associated with, the proliferation, survival, migration/invasion and metastasis of different tumor cells [55,56,57]. It is known that the most important stimulants for the activation of NF-κB are proinflammatory cytokines, like TNFs and IL-1β inducing overexpression of IKK [58,59].

To examine in more detail the signaling pathway of Calebin A’s suppression effects in TNF-β-promoted CRC cells, we evaluated the association of the proinflammatory transcription factor NF-κB signaling pathway in this process. We found, for the first time, that down-modulation of TNF-β-promoted transcription factor NF-κB activation and suppression of its nuclear translocation by Calebin A, and this was also inhibited in the same way by the knockdown of NF-κB on mRNA level using ASO-NF-κB, or on the IKK level by BMS-345541 (specific IKK-inhibitor) or by curcumin (natural inhibitor of NF-κB), may be essential mechanisms for Calebin A’s antitumorigenic potential in this inflammatory tumor environment. Moreover, we have further demonstrated that NF-κB-specific biomarkers, which are linked to invasion (MMP-9), metastasis (CXCR4, Integrin β1), proliferation (Ki-67) and apoptosis (Caspase-3), were modulated by Calebin A. These findings indicate that Calebin A may act as a potent multitargeting anti-cancer agent and this effect is mediated partially through inhibiting NF-κB and NF-κB-promoted proteins in different CRC tumor cells.

Transcription factor NF-κB, which is expressed ubiquitously in the cytoplasm, has been discovered in 1986 and has since been found to be the major regulator of inflammation and tumorigenesis. The constitutive activation of p65-NF-κB (RELA), especially, plays a pivotal role in solid cancers [60]. Phosphorylation of the p65-NF-κB subunit by IKKs/IκBα is a major activation step in the canonical NF-κB pathway, followed by nuclear translocation and DNA binding to modulate gene transcription [61]. Moreover, our findings are also consistent with those that have shown that numerous natural substances mediate their anti-tumorigenic potential through targeting the NF-κB pathway [19,42,62,63,64], thereby stimulating tumor cell apoptosis and suppressing proliferation and migration. Indeed, Calebin A has recently been shown to suppress the activation of the p65-NF-κB pathway in several cell types, thereby suppressing cell growth and enhancing apoptosis [27].

These data emphasize that Calebin A suppresses TNF-β-stimulated tumor-promoting pathways, therefore we focused on investigating the modulatory role of Calebin A on the NF-κB pathway, as this is a major target for TNF-β. Indeed, previous studies have shown that Calebin A inhibits constitutive NF-κB activation as well as negatively regulating TNF-α and LPS-induced NF-κB activation [27]. We could previously show in TNF-β-stimulated CRC cells that NF-κB is a major target for the natural substance resveratrol in suppressing CRC tumor cell malignity [12,13,36,63]. Interestingly, a recent study could show that Calebin A mediates its anti-tumorogenic properties by inhibiting P300/CBP-associated factor (PCAF), which serves as transcriptional co-activator for both p53 and NF-kB [32,65].

In this study, we could further show that Calebin A inhibits TNF-β-up-regulated p65- NF-κB activity in the nucleus of all investigated CRC cell lines. Interestingly, we further observed that Calebin A exerted these effects in CRC cells partly by the direct inhibition of DNA-binding between p65-NF-κB. Previous studies in various tumor cells have shown that, indeed, Calebin A interacts directly with cysteine residues of p65-NF-κB, thereby suppressing the recombinant binding of p65 to DNA [27]. Furthermore, several reports have demonstrated that a residue Cyc38 of p65-NF-κB is essential for the specific binding/interaction to the DNA in different tumor cells [40,43,46,66]. Interestingly, recent studies suggested that polyphenols may mediate their anti-proliferative effects in colon cancer cells through targeting the cyclin-dependent kinase (CDK) activity [67,68]. As the NF-kB family is tightly involved in cell cycle regulation through actions on the CDK/cyclin-dependent kinase inhibitors (CKI) system [69], here, Calebin A, through inhibition of NF-kB DNA binding, may directly suppress cell cycle activation.

## 5. Conclusions

**Figure 7:**
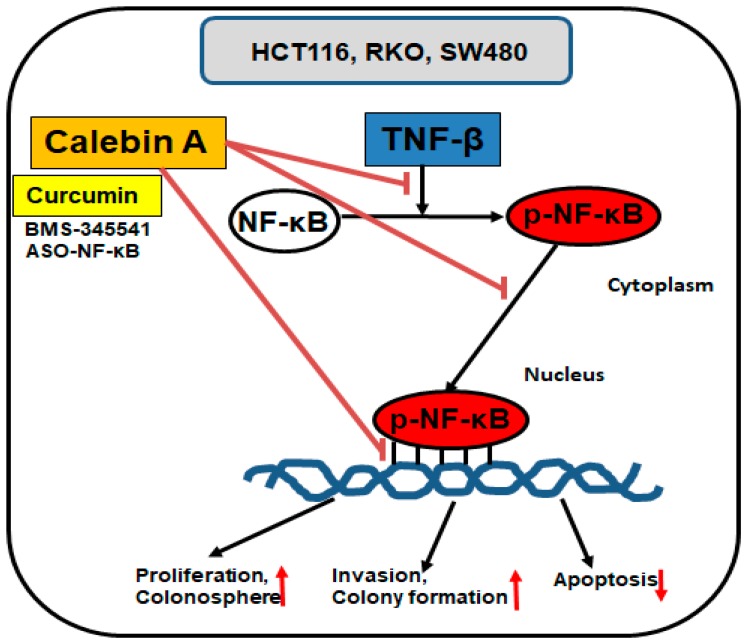
The possible molecular mechanism for the suppression of TNF-β-promoted p65-NF-κB activation during tumorigenesis by Calebin A in vitro.

Conclusively, these findings are the first report demonstrating the potential of Calebin A as an anti-proinflammatory and anti-tumor active compound in the management of TNF-β-stimulated colorectal cancer cell malignity through the targeting and inhibition of activation, nuclear translocation and DNA-binding of p65-NF-κB (Figure 7). These results may play an important role in the preventive therapy of cancer.
